# Genetic monitoring detects an overlooked cryptic species and reveals the diversity and distribution of three invasive *Rattus *congeners in south Africa

**DOI:** 10.1186/1471-2156-12-26

**Published:** 2011-02-16

**Authors:** Armanda D Bastos, Deenadayalan Nair, Peter J Taylor, Helene Brettschneider, Frikkie Kirsten, Elmarie Mostert, Emil von Maltitz, Jennifer M Lamb, Pim van Hooft, Steven R Belmain, Giancarlo Contrafatto, Sarah Downs, Christian T Chimimba

**Affiliations:** 1 Mammal Research Institute, Department of Zoology and Entomology, University of Pretoria, Private Bag 20, Hatfield 0028, Pretoria, South Africa; 2 DST-NRF Centre of Excellence for Invasion Biology (CIB), Department of Zoology & Entomology, University of Pretoria, Pretoria 0002, South Africa; 3 School of Biological and Conservation Sciences, University of KwaZulu-Natal, University Road, Westville, KwaZulu-Natal, 3629, South Africa; 4 Department of Ecology & Resource Management, School of Environmental Sciences, University of Venda, P/Bag X5050, Thohoyandou 0950, South Africa; 5 ARC-Plant Protection Research Institute, Private Bag X134, Queenswood 0121, South Africa; 6 Resource Ecology Group, Wageningen University, Droevendaalsesteeg 3a, 6708 PB Wageningen, The Netherlands; 7 Natural Resources Institute, University of Greenwich, Kent ME4 4TB, UK

## Abstract

**Background:**

South Africa's long and extensive trade activity has ensured ample opportunities for exotic species introduction. Whereas the rich biodiversity of endemic southern African fauna has been the focus of many studies, invasive vertebrates are generally overlooked despite potential impacts on biodiversity, health and agriculture. Genetic monitoring of commensal rodents in South Africa which uncovered the presence of *Rattus tanezumi*, a South-East Asian endemic not previously known to occur in Africa, provided the impetus for expanded studies on all invasive *Rattus *species present.

**Results:**

To this end, intensified sampling at 28 South African localities and at one site in Swaziland, identified 149 *Rattus *specimens. Cytochrome *b *gene sequencing revealed the presence of two *R. tanezumi*, seven *Rattus rattus *and five *Rattus norvegicus *haplotypes in south Africa. Phylogenetic results were consistent with a single, recent *R. tanezumi *introduction and indicated that *R. norvegicus *and *R. rattus *probably became established following at least two and three independent introductions, respectively. Intra- and inter-specific diversity was highest in informal human settlements, with all three species occurring at a single metropolitan township site. *Rattus norvegicus *and *R. rattus *each occurred sympatrically with *Rattus tanezumi *at one and five sites, respectively. Karyotyping of selected *R. rattus *and *R. tanezumi *individuals identified diploid numbers consistent with those reported previously for these cryptic species. Ordination of bioclimatic variables and MaxEnt ecological niche modelling confirmed that the bioclimatic niche occupied by *R. tanezumi *in south Africa was distinct from that occupied in its naturalised range in south-east Asia suggesting that factors other than climate may influence the distribution of this species.

**Conclusions:**

This study has highlighted the value of genetic typing for detecting cryptic invasive species, providing historical insights into introductions and for directing future sampling. The apparent ease with which a cryptic species can become established signals the need for broader implementation of genetic monitoring programmes. In addition to providing baseline data and potentially identifying high-risk introduction routes, the predictive power of ecological niche modelling is enhanced when species records are genetically verified.

## Background

The long and close association of members of the genus *Rattus *(Fischer de Waltheim) and *Mus *(Linnaeus) with humans have, primarily due to their high levels of adaptability and small size, made them two of the most successful invasive mammalian genera. Whilst the negative impacts of commensal rodents, and *Rattus *in particular, on humans with respect to health and competition for food are undisputed [1], the positive contribution that some species have made as model/experimental animals in biomedical research [2], and more recently towards tracing historical human migrations [3,4], are increasingly acknowledged.

When introduced to areas outside their native range, the effects of invasive rodents on endemic biodiversity and on health can be detrimental, particularly in island ecosystems. As reservoirs of a wide range of pathogens [5] capable of infecting diverse hosts, they have the potential to cause localised die-offs in native large mammal species [6] and in at least one extreme case, are believed to have precipitated the extinction of two endemic rats, *R. macleari *and *R. nativitatus *on Christmas island [7]. Their introduction to any ecosystem should, therefore, be carefully monitored to identify and address their presence and in so-doing, limit their impact. This hinges on early detection, which can be difficult particularly in the case of *Rattus*, a diverse genus containing numerous cryptic species. As a number of *Rattus *species have become successfully established outside their native range, this situation lends itself to a 'silent invasion' whereby a species can potentially go unnoticed for an extended period of time if its introduction was preceded by a cryptic species. This is partly because despite the substantive and undeniable impacts of invasive *Rattus *species, and their almost worldwide distribution, there is a lack of definitive morphological keys due to the paucity of studies on the taxonomy, origin and natural history of members of this globally-distributed invasive genus. A recent attempt to refine the higher-level taxonomy of the group and to base classifications within the Rattini tribe on a systematic approach that is firmly grounded in genetics [8] has highlighted the value of molecular phylogenetic approaches in addressing the intrinsic difficulties of this group of animals [1]. The study revealed the presence of at least seven discrete monophyletic lineages within the Indochinese *Rattus rattus *species complex, two more than predicted from traditional taxonomy, and also identified numerous examples of morphological misidentification [8]. In addition to delimiting species and resolving taxonomic relationships [8], molecular data permit estimations of the sequence and timing of species divergence. The generation of complete mitochondrial genome sequences of wild-caught species has provided more accurate estimations of the timing of divergences of five *Rattus *congeners [9], namely; the black rat, *R. rattus *(Linnaeus), the brown rat, *R. norvegicus *(Berkkenhaut), the Asian house rat, *R. tanezumi *(Temminck), the Polynesian rat, *R. exulans *(Peale) and the spiny rat, *R. praetor *(Thomas). Detailed intra-specific studies such as those performed on the Pacific rat, *R. exulans*, indicate that this species can be used as a proxy for studying pre-historic human migrations [10] and that *R. rattus *may similarly provide insights into more recent human migrations [4]. Despite its widespread occurrence, the available molecular and genetic data for *R. norvegicus *are primarily restricted to laboratory-adapted strains [2,11] and data for wild-caught animals remains limited. Thus although invasive *Rattus *species are receiving increased attention, the complex taxonomy of the genus which is characterised by difficulties in morphology-based identifications, remains unclear [1,12,13,8] and there is a paucity of data from areas invaded by members of this genus which comprises *ca*. 65 species. The availability of a comprehensive, homologous dataset from throughout the invaded and native ranges of each species would provide insights into the origins and dispersal routes of these economically important commensal species.

South Africa's rich biodiversity is exemplified by the approximately 50 murid rodent species which are known to occur in this country [14]. Only a small proportion of species, notably members of the genera *Rattus*, *Mastomys*, and *Gerbilliscus *(formerly *Tatera*; [15]), have been implicated in disease transmission [16,17] and in causing damage to crops and stored grain [18]. The presence of cryptic species in South Africa and elsewhere [19,20], complicates control and the value of molecular approaches for identifying morphologically indistinguishable murid rodents is increasingly being recognised [21,13,8]. This approach was applied in a European Commission and DFID-funded community participatory research programme undertaken in South Africa. It was during this routine molecular monitoring that the presence of *R. tanezumi *was discovered in South Africa [21,17], representing the first records of this species on the African continent. Before this, just two invasive *Rattus *species, *R. norvegicus *and *R. rattus*, were known to occur in South Africa [22]. The unexpected presence of a third *Rattus *species, which together with its sibling species, *R. rattus *forms part of the *R. rattus *species complex, provided the impetus for further sampling and genetic characterisation of rats from four provinces in South Africa, and forms the basis for this study, which aimed to determine the genetic diversity and geographic distribution of invasive, commensal *Rattus *species in South Africa. To this end, 149 *Rattus *specimens from South Africa and Swaziland were identified to species level on the basis of mitochondrial cytochrome *b *(cyt *b*)gene sequencing. Karyotyping of selected *R. rattus *and *R. tanezumi *individuals was performed to confirm the molecular identifications. Geographic distribution records for the native range of *R. tanezumi *(obtained from public domain museum databases provided by the Global Biodiversity Information Facility; http://www.gbif.org) were used to predict the distribution of this species in sub-Saharan Africa and to determine the factors that may influence its distribution.

## Methods

### Species and sampling

Two invasive *Rattus *species with markedly different geographical distributions occur in South Africa: *R. norvegicus *is reported to be a coastal-bound species and *R. rattus *as being widely distributed throughout the country [22]. A third, newly recorded species, *R. tanezumi *was identified from two sites in Limpopo Province [21] and from one site in KwaZulu-Natal Province [17]. The subsequent intensified sampling, confirmed *Rattus *species presence at two further Limpopo Province (LP) localities, at one site in Mpumalanga Province (MP), eight in Gauteng Province (GP), 15 in KwaZulu-Natal (KZN) Province and one site in Swaziland (summarised in Tables 1 and 2). In this study, 'south Africa' refers to the region that incorporates South Africa and the two landlocked countries of Lesotho and Swaziland.

**Table 1:** Summary of the cytochrome *b *gene sequences generated in this study for the three invasive *Rattus *species occurring in South Africa and the reference sequences used for genetic analyses

*Rattus tanezumi *(south Africa)				
**Haplotype**	**Sampling Locality**	**N**	**Genbank AN (Museum AN)**	**Reference**
RT01	Giyani (Nkomo-B), LP	7	DQ439816- DQ439822	This study
RT01	Tshilimbani, LP	19	HQ157805	This study
RT01	Tholakele, Paulpietersburg, KZN	2	DQ439823, DQ439826	This study
RT01	Mvuzini, Vryheid, KZN	3	DQ439824-5, DQ439827	This study
RT01	Ophuzane, Paulpietersburg, KZN	6	DQ439829, DQ439845-8	This study
RT01	Hammanskraal, GP	12	DQ439828	This study
RT01	Rietondale, Pretoria, GP	1	DQ444864	This study
RT01	Moreleta Park, Pretoria, GP	1	FJ842262	This study
RT01	Tembisa, GP	3	HQ157804	This study
RT01	Roodeplaat, GP	13	HQ157807	This study
RT01	Richmond, KZN	1	FJ842263 (DM8685)	This study
RT01	Shongweni, Durban, KZN	2	DQ439849-50 (DM8401, DM8402)	This study
RT01	Swaziland (Mcaphozini Area)	6	FJ842264 (DM8892, DM8896-8900)	This study
RT02	Tembisa, GP	1	FJ842265	This study
**2**	**14 localities**	**77**		

***Rattus tanezumi *** **(elsewhere)**				

**Haplotype**	**Country (Locality)**	**N**	**Genbank AN (Museum AN)**	**Reference**
RT03	Japan, Vietnam	3	AB355899-900, AB211040	[29,30]
RT04	Japan (Amami Island)	1	EU273712	[9]
RT05	Japan	1	AB211041	[29]
RT06	Vietnam	1	AB355901	[30]
RT07	China	1	AB096841	[28]
**5**	**3 countries**	**7**		

***Rattus rattus *** **(South Africa)**				

**Haplotype**	**Sampling Locality**	**N**	**Genbank AN (Museum AN)**	**Reference**
RR01	Giyani (Nkomo-B), LP	9	DQ439830, DQ439851-8	This study
RR01	Sekhukhune (Bloublommetjieskloof), LP	2	DQ439831-2	This study
RR01	Tshilimbani, LP	4	HQ157802	This study
RR01	Tembisa, GP	1	HQ157801	This study
RR03	Hammanskraal, GP	4	DQ439834-5	This study
RR03	Moreleta Park, Pretoria, GP	1	FJ842266	This study
RR03	Tembisa, GP	2	HQ157800	This study
RR03	Verena, MP	1	HQ157803	This study
RR04	Renosterkop, LP	1	HQ157806	This study
RR04	Hammanskraal, GP	1	DQ439833	This study
RR04	Hillcrest, Pretoria, GP	1	FJ842267	This study
RR04	Umkomaas, KZN	5	DQ439836-8 (DM8403-7)	This study
RR05	Hammanskraal, GP	1	FJ842268	This study
RR06	Cape Town, WCP	2	GQ891608	[4]
RR10	Melmoth, KZN	1	[HQ157808] (DM8820)	This study
RR11	Ladysmith, KZN	1	[HQ157809] (DM8907)	This study
**7**	**13 localities**	**37**		

***Rattus rattus *** **(elsewhere)**				

**Haplotype**	**Country (Locality)**	**N**	**Genbank AN**	**Reference**
RR01	Tanzania	1	H217365	[8]
RR02	Indonesia	1	AB033702	[27]
RR06	New Zealand (Titirangi)	1	EU273707	[9]
RR07	Japan	2	AB211039 [AB211042]	[29]
RR08	India	1	H217367	[8]
RR09	Oman	1	H217366	[8]
**6**	**6 countries**	**7**		

***Rattus norvegicus *** **(South Africa)**				

**Haplotype**	**Sampling Locality**	**N**	**Genbank AN (Museum AN)**	**Reference**
RN01	Sydenham, Durban, KZN	2	FJ842270 (DM7744, DM7745)	This study
RN01	Durban CBD, KZN	3	FJ842271 (DM7783, DM7804-5)	This study
RN01	Montclair Park, Durban, KZN	2	DQ439842-3 (DM7788, DM7789)	This study
RN01	Cato Crest IS, Durban, KZN	2	FJ842272 (DM7802, DM7803)	This study
RN01	Cato Manor Rd, Durban, KZN	1	DQ439844 (DM7820)	This study
RN01	Tembisa, GP	1	FJ842269	This study
RN01	O.R. Tambo International Airport, GP	1	FJ842273	This study
RN01	Johannesburg Zoological Gardens	8	HQ157799	This study
RN02	Cato Crest IS, Durban, KZN	2	FJ842274 (DM7790, DM7791)	This study
RN03	Durban Harbour, KZN	2	DQ439840-1 (DM7706, DM7711)	This study
RN04	Warwick Ave, Durban, KZN	1	DQ439839 (DM7645)	This study
RN12	O.R. Tambo International Airport, GP	9	FJ842275	This study
RN12	Tembisa, GP	3	FJ842276	This study
**5**	**10 localities**	**37**		

***Rattus norvegicus *** **(elsewhere)**				

**Haplotype**	**Sampling Locality of wild caught animals/(Lab Strain)**	**N**	**Genbank AN**	**Reference**
RN01	(BN/SsNHsdMCW)	1	AY172581	[46]
RN01	China*, Sweden	2	[GU592972*], FJ919765	[11]
RN09	(GH/OmrMcwi), [GH/Swe]	2	DQ673911, [DQ673913]	[2]
RN10	Milwaukee, USA	1	DQ673916	[2]
RN05	Indonesia	1	FJ842279	This study
RN07	Vietnam	1	FJ842278	This study
RN08	Vietnam	2	AB355902, [AB355903]	[30]
RN11	Copenhagen, Denmark	1	AJ428514	[47]
RN06	Vietnam	1	FJ842277	This study
RN13	Tokyo, Japan	1	DQ673917	[2]
RN13	(T2DN/Mcwi)	1	DQ673915	[2]
RN14	(Sprague-Dawley)	1	AB033713	[48]
RN15	(Wistar)	1	X14848	[49]
**11**	**7 countries**	**16**		

AN: Accession Number; LP: Limpopo Province; GP: Gauteng Province; KZN: KwaZulu-Natal Province; MP: Mpumalanga Province; WCP: Western Cape Province; CBD: Central Business District; IS: Informal Settlement; DM: Durban Natural Science Museum; []: Sequences that were not used for phylogenetic inference; * Unpublished record.

**Table 2:** Summary of the 149 genetically characterised *Rattus *specimens sampled from 29 localities in south Africa.

Localities	GPS	Metres a.s.l.	N	N (per species)	Haplotypes
Giyani (Nkomo-B), LP	23.416S - 30.786E	448 m	16	*R. tanezumi *(7)	RT01*
				*R. rattus *(9)	RR01*
Renosterkop, LP	24.983S - 28.525E	1050 m	1	*R. rattus *(1)	RR04
Tshilimbani, LP	22.767S - 30.200E	863 m	23	*R. tanezumi *(19)	RT01
				*R. rattus *(4)	RR01
Sekhukhune (Bloublommetjieskloof), LP	24.311S - 29.770E	743 m	2	*R. rattus *(2)	RR01
Johannesburg Zoological Gardens, GP	26.166S - 28.037E	1620 m	8	*R. norvegicus *(8)	RN01
Moreleta Park, Pretoria, GP	25.828S - 28.288E	1545 m	2	*R. tanezumi *(1)	RT01*
				*R. rattus *(1)	RR03
Roodeplaat, GP	25.639S - 28.358E	1225 m	13	*R. tanezumi *(13)	RR01
Rietondale, Pretoria, GP	25.731S - 28.219E	1313 m	1	*R. tanezumi *(1)	RT01
Hillcrest, Pretoria, GP	25.754S - 28.231E	1380 m	1	*R. rattus *(1)	RR04
Hammanskraal, GP	25.371S - 28.188E	1105 m	18	*R. tanezumi *(12)	RT01
				*R. rattus *(4)	RR03*
				*R. rattus *(1)	RR04*
				*R. rattus *(1)	RR05
Tembisa, GP	26.002S - 28.213E	1605 m	11	*R. tanezumi *(3)	RT01
				*R. tanezumi *(1)	RT02
				*R. rattus *(1)	RR01
				*R. rattus *(2)	RR03
				*R. norvegicus *(1)	RN01
				*R. norvegicus *(3)	RN12
O.R. Tambo International Airport, GP	26.148S - 28.226E	1693 m	10	*R. norvegicus *(1)	RN01
				*R. norvegicus *(9)	RN12
Verena, MP	25.504S - 29.027E	1369 m	1	*R. rattus *(1)	RR03
Tholakele, KZN	27.434S - 30.988E	1100 m	2	*R. tanezumi *(2)	RT01
Mvuzini, KZN	28.008S - 30.675E	1172 m	3	*R. tanezumi *(3)	RT01
Ophuzane, KZN	27.486S - 30.934E	827 m	6	*R. tanezumi *(6)	RT01
Richmond, KZN	29.883S - 30.283E	860 m	1	*R. tanezumi *(1)	RT01*
Melmoth, KZN	28.567S - 31.317E	980 m	1	*R. rattus *(1)	RR10
Ladysmith, KZN	28.550S - 29.783E	1047 m	1	*R. rattus *(1)	RR11
Shongweni, Durban, KZN	29.834S - 30.699E	446 m	2	*R. tanezumi *(2)	RT01*
Umkomaas, KZN	30.217S - 30.800E	14 m	5	*R. rattus *(5)	RR04*
Sydenham, Durban, KZN	29.832S - 31.000E	52 m	2	*R. norvegicus *(2)	RN01
Durban CBD, KZN	29.859S - 31.016E	22 m	3	*R. norvegicus *(3)	RN01
Montclair Park, Durban, KZN	29.925S - 30.965E	33 m	2	*R. norvegicus *(2)	RN01
Cato Manor Rd, Durban, KZN	29.858S - 30.978E	88 m	1	*R. norvegicus *(1)	RN01
Cato Crest IS, Durban, KZN	29.850S - 30.978E	88 m	4	*R. norvegicus *(2)	RN01
				*R. norvegicus *(2)	RN02
Durban Harbour, KZN	29.871S - 31.048E	6 m	2	*R. norvegicus *(2)	RN03
Warwick Ave, Durban, KZN	29.858S - 31.010E	22 m	1	*R. norvegicus *(1)	RN04
Manzini, Mcaphozini Area, Swaziland	26.435S - 31.197E	679 m	6	*R. tanezumi *(6)	RT01

a.s.l.: above sea level; LP: Limpopo Province, South Africa; GP: Gauteng Province, South Africa; KZN: KwaZulu-Natal Province, South Africa; IA: International Airport; CBD: Central Business District; IS: Informal Settlement; * indicates haplotypes that were karyotyped

Samples were obtained by snap-trapping and through routine extermination programmes conducted by pest control companies in townships and at facilities such as the O.R. Tambo International Airport. A small number of animals were live-trapped, using Sherman traps (H.B. Sherman Traps Inc. Florida, U.S.A.) baited with a mixture of peanut butter, oatmeal and fish oil. After capture, during transportation and in the laboratory, the live-trapped animals were kept in polyurethane cages with wood shavings provided as bedding, and mouse pellets and water provided *ad libitum*, as per the guidelines of the American Society of Mammalogists (ASM; http://www.mammalogy.org/committees/index.asp; Animal Care and Use Committee 1998). Animals were euthanized in accordance with the protocol approved by the Animal Ethics subcommittee of the University of KwaZulu-Natal Research Committee. Voucher specimens were prepared using standard natural history museum procedures for mammal specimens and are deposited in the South African mammal reference collections of the Transvaal Museum (TM) of the Northern Flagship Institute (NFI) in Pretoria and the Durban Natural Science Museum (Table 1).

### Cytochrome *b *gene characterisation

Total genomic DNA was extracted from either liver or kidney samples using the High Pure PCR template preparation kit (Roche) according to the prescribed manufacturer protocol. The entire cytochrome *b *gene was amplified using murid rodent primers that bind within the mitochondrial genome regions flanking cyt *b*, namely L14724 TGAYATGAAAAAYCATCGTTG and H15915 CATTTCAGGTTTACAAGAC [20]. The thermal cycling profile consisted of two cycles of denaturation at 96°C for 12 s, primer annealing at 52°C for 30 s, and extension at 70°C for 60 s. Keeping denaturation and extension steps the same, the next three cycles were performed at an annealing temperature (Ta) of 50°C, with the Ta of the final 35 cycles being adjusted to 48°C. The resulting amplicon, approximately 1.2 kb in length, was purified directly from the tube using the High Pure PCR product purification kit (Roche) according to manufacturer specifications. Nucleotide sequences were determined by cycle sequencing with either version 3.0 or 3.1 of the Big Dye Terminator Cycle Sequencing Ready Reaction kit (Applied Biosystems), at a Ta of 48°C, with each of the PCR primers in separate reactions. Sequences were precipitated and run on the ABI PRISM™ 3100 Analyser (Applied Biosystems). Forward and reverse nucleotide sequences were viewed and aligned in Mega4 [23] and full-length cyt *b *gene contigs, 1140 nucleotides (nt) in length, were generated for 147 southern African *Rattus *specimens and for three additional samples obtained from Indonesia and Vietnam (Table 1). Two partial sequences were also generated for *R. rattus *haplotypes RR10 and RR11 from KZN (Table 1). Of the 152 *Rattus *sequences generated, 143 were from *Rattus *sampled in South Africa (71 *R. tanezumi*, 35 *R. rattus *and 37 *R. norvegicus*), six were from Swaziland and three from outside south Africa (Table 1). At least one sequence per species, locality and haplotype was submitted to Genbank http://www.ncbi.nlm.nih.gov under the accession numbers listed in Table 1.

### Molecular analyses

The 150-taxon full-length *Rattus *cyt *b *gene dataset generated in this study, and containing specimens from South Africa, Swaziland, Indonesia and Vietnam, was reduced to a dataset of 24 taxa which retained all of the sequence diversity on a per-species, per-province and per-haplotype basis. This dataset was complemented with 23 full-length cyt *b *gene sequences containing 20 *Rattus *conspecific sequences available in the Genbank database (Table 1) and three *Mus musculus *outgroup sequences. The latter comprised two Genbank entries (accession numbers EF108343-4) and one *M. m. domesticus *haplotype identified in this study following nucleotide sequencing of two specimens from South Africa (accession number HQ157798). The best-fit model and parameters identified under the Akaike Information Criterion (AIC) in ModelTest 3.7 [24] guided model selection for the Minimum Evolution (ME) and Maximum Likelihood (ML) analyses performed in Mega4 [23] and PhyML [25], respectively, and for setting priors for Bayesian inference (BI) with MrBayes [26]. For the latter analyses, four Markov Monte Carlo chains were run for 10,000,000 generations using default heating and swap settings, and were sampled every 2000 generations, with 20% of the run being discarded as 'burn-in'. Three independent runs were performed to ensure convergence. Nodal support for clades was assessed by 100,000 and 1,000 non-parametric bootstrap replicates for ME and ML, respectively, and from Bayesian posterior probabilities (BPP). In addition to inferring relationships from available full-length cyt *b *sequences, partial cyt *b *datasets (> 1000 bp in length) were compiled for *R. rattus *and *R. tanezumi *which incorporated the haplotype diversity reported for these species in previous studies [27,9,30,8,4].

Nucleotide and haplotype diversity indexes were determined in DNAsp version 5 [31]. Median-joining networks were constructed for each *Rattus *species in Network 4.5.1.0 [32] which takes pairwise haplotype differences into account to generate a network of parsimonious relationships that combines all possible trees using a one-step mutation model [33]. For *R. rattus *and *R. tanezumi*, haplotype networks were constructed from full-length as well as the partial cyt *b *datasets. All *R. norvegicus *analyses were based on full-length gene sequences.

### Karyotyping

Eight specimens typed as *R. rattus *and five as *R. tanezumi *following cyt *b *sequencing were selected for karyotyping (Table 2). Metaphase chromosome spreads were prepared from either bone marrow samples flushed from the shafts of the tibiae and/or femorae of each sacrificed specimen as originally described by Hsu and Patton [34], or from skin fibroblast cultures established from < 1 cm^2 ^ear biopsies, as described by Contrafatto *et al*. [35]. Giemsa-stained metaphase plates with the best morphology and least chromosomal overlaps were digitally recorded using a Vanox AHBS3 Olympus compound microscope connected to a coupled charged device (CCD) video camera (COHU, Japan), linked in turn to an Intel-based personal computer equipped with a miroVideo PCTV capture board (Pinnacle Systems, Germany). Digitised metaphase spreads were used to construct at least two karyograms per specimen using the open-source package Gimp, version 1.2.3 http://www.gimp.org. The diploid number was determined by establishing the modal number of chromosomes from chromosome counts of no less than 20 metaphase spreads per individual, following the guidelines of Yosida [36] for *Rattus *karyogram construction using uniformly stained chromosomes.

### Ecological niche modeling

Notwithstanding acknowledged limitations, bioclimatic data from the native range ("niche") of an invasive species can be used to predict the extent of invasion in a target area [37-40]. This approach assumes climatic matching of native and target areas. To test this assumption, using principal components analysis (PCA), we compared the bioclimatic space occupied by 13 geo-referenced *R. tanezumi *distribution records from the invasive range of the species in South Africa and Swaziland (precision of 0.001 degrees) with that obtained from 134 unique-locality occurrence records from the combined naturalised and native range extracted from public domain databases provided by the Global Biodiversity Information Facility http://www.gbif.org. Altitude and eight bioclimatic variables (WORLDCLIM version 1.4: http://www.worldclim.org/bioclim; [41]) were originally chosen (see additional file 1, Table S1), reflecting means, extremes, and seasonal variation of temperature and precipitation, viz. Bio1 (mean annual temperature), Bio4 (temperature seasonality), Bio5 (maximum temperature of warmest month), Bio6 (minimum temperature of coldest month), Bio12 (annual precipitation), Bio13 (precipitation of wettest month), Bio14 (precipitation of driest month), and Bio15 (precipitation seasonality). In order to compare the relative importance of human and climate variables in the respective ecological niches of the species from its invasive and naturalised ranges, "human footprint", a global map of human influence on the landscape was also used [42].

Next we estimated the ecological niche for *R. tanezumi *based on 119 native and naturalised occurrence records, in order to predict the species' potential invasive distribution in sub-Saharan Africa, and to compare this with the actual known invasive range. We used the MaxEnt (Maximum Entropy; [43]) programme which employs a general machine learning algorithm, because it has been shown to perform well with presence-only data and with small sample sizes, and generally outperforms alternative "climatic envelope" models such as GARP and BIOCLIM [44]. To correct for potential statistical over-fitting due to high correlation coefficients between certain bioclimatic variables, we summarised the pattern of correlations between the original set of eight bioclimatic variables by means of an Unweighted Pair-Group Method with Averages (UPGMA) correlation phenogram. Individual variables were selected from four distinct (independent) clusters (data not shown): Bio1 (mean annual temperature), Bio4 (temperature seasonality), Bio12 (annual precipitation), Bio14 (precipitation of driest month). Given the known commensalism of rats, including *R. tanezumi*, and following Ficetola *et al*. [38] "human footprint" was included as a fifth variable.

The environmental data were set to a spatial grid resolution of 2.5 arc minutes and were clipped to an area accommodating the native and naturalised ranges of *R. tanezumi*. The projection layer (target area) was clipped to sub-Saharan African south of 13 degrees north. The MaxEnt model was run with 70% presence records used for training and 30% for random testing, with the regularization multiplier set to 1.0, maximum number of iterations to 1500, convergence threshold to 1 × 10-5 and output format to logistic. Duplicate records (in the same pixel) were excluded. Model performance was assessed with proportion of presences correctly classified (sensitivity), proportion of absences correctly classified (specificity), and discrimination ability (area under the curve [AUC] of a receiver operating characteristic [ROC] plot of sensitivity versus 1-specificity). Since MaxEnt produces a continuous probability (ranging from 0 to 1.0), the continuous model output was transformed to a map representing probabilities of occurrence. The contribution of each explanatory variable to model performance was evaluated with a jackknife procedure implemented in MaxEnt, where variables are successively omitted and then used in isolation to measure their relative, as well as their absolute contribution to the model.

## Results

### Genetic analyses

The 44-taxon *Rattus *ingroup dataset contained 183 variable and 165 parsimony-informative sites across the 1140 nucleotide (nt) cyt *b *gene region. As would be expected for a coding gene, the proportion of base position mutations was 3^rd ^> 1^st ^> 2^nd^, with 138 (75%) of the mutations occurring in the 3^rd ^base position, 39 (21%) in the 1^st ^base position and the remaining 6 (4%) being attributed to 2^nd ^base position mutations.

**Figure 1:**
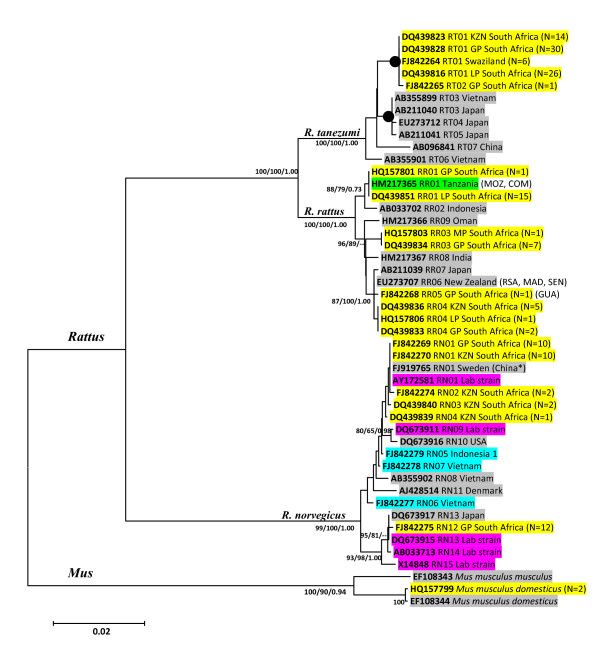
**Cytochrome *b *gene tree inferred using full-length sequence data (1140 bp) and depicting the genetic relatedness of the three *Rattus *species haplotypes in South Africa**. Taxon names comprise the Genbank accession number, followed by the country of origin (for South African samples this is preceded by a province code: LP = Limpopo Province, MP = Mpumalanga Province, GP = Gauteng Province, KZN = KwaZulu-Natal), haplotype and the number of individuals characterised. Nodal support values are given in percentages and are indicated ME/ML/BPP next to the relevant nodes. -- indicates nodes that were either not recovered or that had support values < 50 for ME and < 70 for ML and BPP. Haplotypes are colour-coded as follows to indicate the region and source of the data: Yellow = Southern Africa (This study), Blue = Outside Africa (This study), Green = Africa (Genbank), Grey = Outside Africa (Genbank), Purple = Laboratory strain (Genbank). Terminal nodes connecting different haplotypes and having ≥99 percent support from all three methods of inference are denoted by a black-filled circle. Country codes given in brackets behind a taxon name, indicate shared presence of a particular haplotype identified from the partial 1043 bp dataset (additional file 3, Figure S3) and are abbreviated: COM = Comores, MOZ = Mozambique, GUA = Guadeloupe, MAD = Madagascar, RSA = Republic of South Africa and SEN = Senegal.

**Figure 2:**
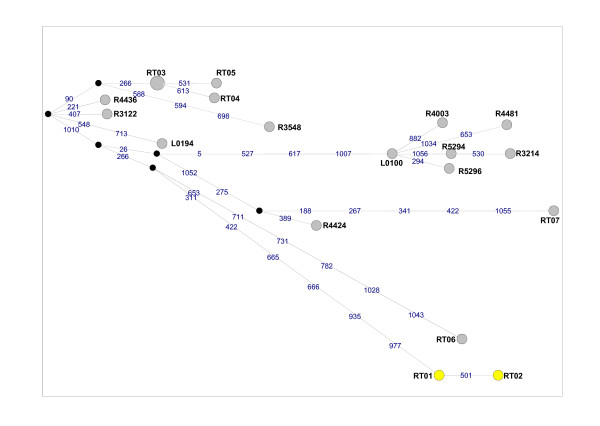
**Median-joining network of *Rattus tanezumi *cytochrome *b *(cyt *b*) haplotypes based on partial (1077 bp) gene sequences**. Haplotype colour coding is consistent with that used in Figure 1, *viz*. Yellow = south Africa (This study), Grey = Outside Africa (Genbank). Each of the mutational steps separating haplotypes is indicated in blue and corresponds to the relevant position in the cyt *b *gene, whilst black nodes correspond to median vectors. Haplotype numbers are the same as those provided in Table 1. Additional haplotype numbers correspond to the laboratory numbers assigned in the Pagès et al. [8] study.

**Figure 3:**
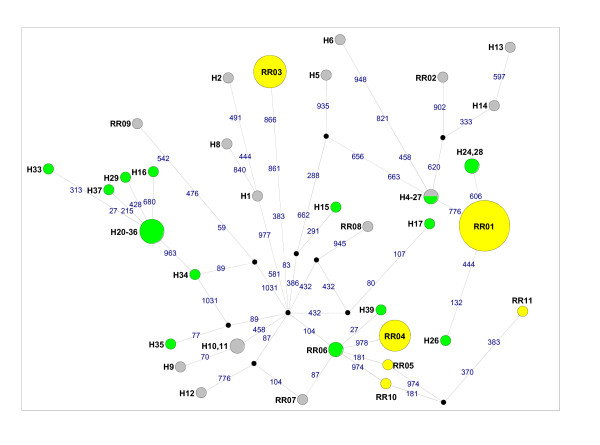
**Median-joining network of *Rattus rattus *cytochrome *b *(cyt *b*) haplotypes based on partial (1043 bp) gene sequences**. The circle size of south African *Rattus *haplotypes is proportional to the frequency of the haplotype. Haplotype colour coding is consistent with that used in Figure 1, *viz*. Yellow = south Africa (This study), Blue = Outside Africa (This study), Green = Africa (Genbank), Grey = Outside Africa (Genbank). Each of the mutational steps separating haplotypes is indicated in blue and corresponds to the relevant position in the cyt *b *gene, whilst black nodes correspond to median vectors. Haplotype numbers are the same as those provided in Table 1. Additional haplotype numbers correspond to the laboratory numbers assigned in the Tollenaere et al. [4] study.

**Figure 4:**
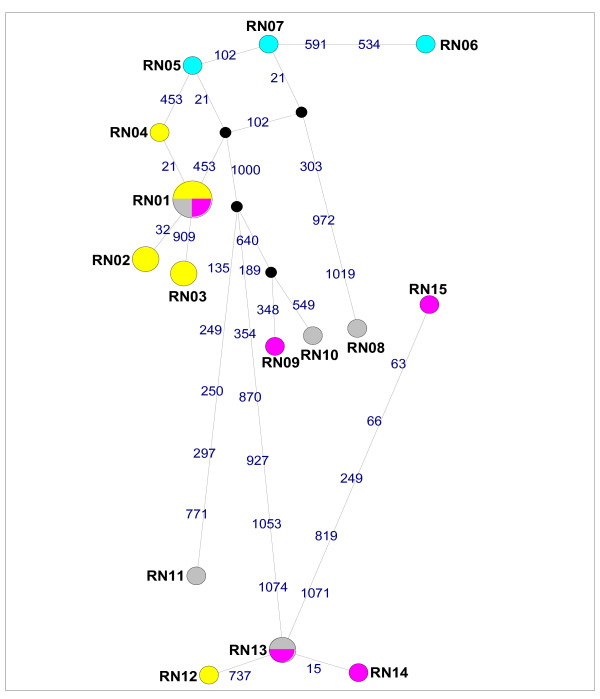
**Median-joining network of *Rattus norvegicus *cytochrome *b *(cyt *b*) haplotypes based on full-length gene sequences**. The circle size of south African *Rattus *haplotypes is proportional to the frequency of the haplotype. Haplotype colour coding is consistent with that used in Figure 1, *viz*. Yellow = south Africa (This study), Blue = Outside Africa (This study), Grey = Outside Africa (Genbank), Purple = Laboratory strains (Genbank). Each of the mutational steps separating haplotypes is indicated in blue and corresponds to the relevant position in the cyt *b *gene, whilst black nodes correspond to median vectors. Haplotype numbers are the same as those provided in Table 1.

For the 47-taxon dataset, inclusive of three *Mus *outgroup sequences, the GTR+I+G model (I = 0.5084 and G = 0.8058) with nucleotide frequencies of A = 0.308, C = 0.288, G = 0.123, T = 0.281, was selected as the best-fit model under the AIC in ModelTest. The cyt *b *gene tree (Figure 1) recovered three monophyletic *Rattus *lineages in south Africa. Nodes that had ≥ 70% bootstrap support in the phenetic analyses generally had high levels of support in the maximum likelihood analyses (Figure 1). The two South African *R. tanezumi *haplotypes, RT01 and RT02, formed a monophyletic lineage (100% support; Figure 1) and differed from each other at just one nucleotide site at position 538 in the full-length dataset (see additional file 2, Figure S2a) and position 501 in the partial cyt *b *dataset (Figure 2). This first base mutation results in a non-synonymous alanine to threonine amino acid substitution. For *R. rattus*, three discrete lineages were recovered for the South African specimens (Figure 1). Haplotype RR01, which occurred in 16 individuals sampled at four South Africa sites, was also present in an individual from Tanzania (Tables 1 & 2). The RR01 haplotype, was sister to haplotype RR02 from Indonesia (73-88% nodal support, Figure 1) differing from it by four mutational steps (see additional file 2, Figure S2b). Haplotypes RR04 and RR05 clustered within a lineage containing representatives from New Zealand (RR06) and Japan (RR07), with high levels of support (87-100% nodal support, Figure 1). The third South African haplotype lineage, RR03, is separated from the former South Africa-New Zealand-Japan cluster by at least six mutational steps (Figure 3), and unrelated to all other haplotypes included in the full-length (Figure 1) and partial dataset (see additional file 3, Figure S3) analyses. For *R. norvegicus*, two discrete South African lineages were recovered. Three of the five South African haplotypes (RN02, RN03 and RN04) were linked to haplotype RN01, recovered from 20 South African specimens (Figure 4), which was also represented by laboratory strain, BN/SsNHsdMCW (Genbank accession number AY172581; Figure 1). Haplotype RN12 which represents the second *R. norvegicus *lineage in South Africa, predominated at two Gauteng Province sites, and was sister to RN13, a haplotype present in the T2DN/Mcwi lab strain and in a wild-caught individual from Japan (Figure 1), differing from the latter two sequences at a single nucleotide site (Figure 4).

Haplotype diversity (h) and nucleotide diversity (π) values determined from complete gene sequence datasets for each of the three south African *Rattus *species were: (i) *R. norvegicus*: h = 0.613 (5 haplotypes from 37 sequences), π = 0.00379, (ii) *R. tanezumi*: h = 0.026 (2 haplotypes from 77 sequences), π = 0.00002, and (iii) *R. rattus*: h = 0.667 (4 haplotypes from 33 sequences), π = 0.00514. Between-species mean p-distance calculated in Mega4 [23] was 3.9% for the two sibling species of the *R. rattus *species complex, *R. tanezumi*-*R. rattus*, 11.9% for *R. tanezumi*-*R. norvegicus *and 11.2% for *R. rattus*-*R. norvegicus*.

**Figure 5:**
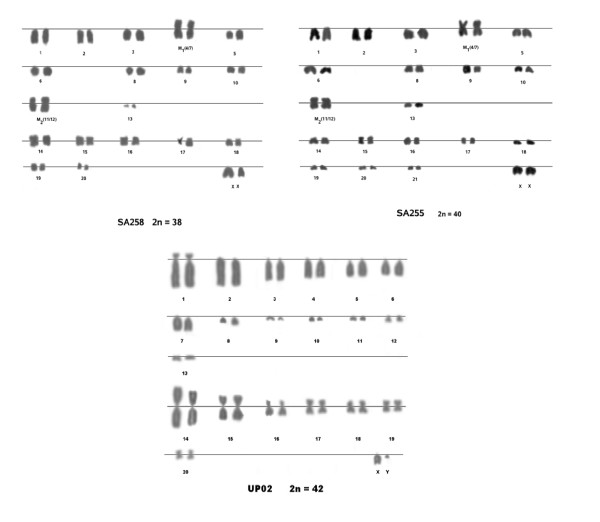
***Rattus *species karyotypes from South Africa**. *Rattus rattus *(haplotype RR04, *2n *= 38) and *Rattus rattus *(haplotype RR04, *2n *= 40) above, with *Rattus tanezumi *(haplotype RT01, *2n *= 42) depicted below.

Karyotyping of eight *R. rattus *individuals recovered diploid chromosome numbers of 38 in six specimens and 40 in two specimens (Figure 5). All five *R. tanezumi *specimens had the *2n *= 42 karyotype that is characteristic of this species (Figure 5). Two of the *R. rattus *individuals (SA255 and SA258) which were identical across the cyt *b *gene (both haplotype RR04) and which were sampled from the same locality (Umkomaas, KwaZulu-Natal Province), were found to have different chromosome numbers (Figure 5). The *R. rattus 2n *= 38 arrangement was characterized by nine pairs of acrocentric chromosomes, metacentric M_1 _and M_2 _(marker) chromosomes, seven additional pairs of metacentric chromosomes and one pair of acrocentric X chromosomes, conforming precisely to the "Oceania" *R. rattus *arrangement described by Yosida *et al*. [45]. Marker chromosomes M_1 _and M_2 _represent centric fusion events involving chromosomes 4/7 and 11/12 respectively of the putative ancestral karyotype [45]. The *2n *= 40 arrangement was identical to that of the *2n *= 38, except that it contained an additional pair (pair 21) derived from a centric fission event. In this respect, this arrangement represents an intermediate stage between the Oceania form and the "Mauritian" form [45] which was derived from the *2n *= 38 form by two centric fission events, and which has not been documented to date. The *2n *= 42 arrangement matched *R. tanezumi *[45] and was characterized by 13 acrocentric chromosome pairs, seven pairs of metacentric chromosomes and acrocentric X and Y chromosomes. No marker chromosomes were identified for this arrangement (Figure 5).

### Geographical distribution

**Figure 6:**
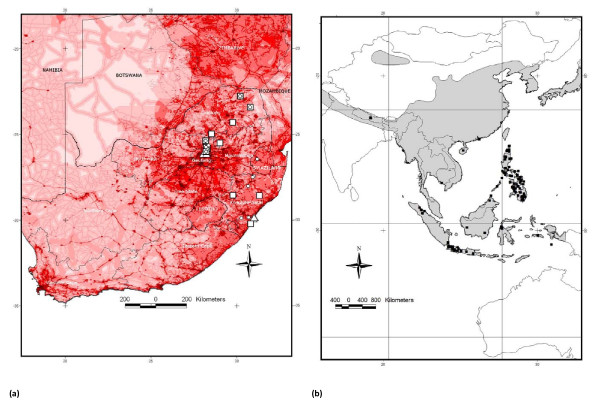
***Rattus *distribution in south Africa and in south-east Asia**. (a) Sampling sites of the three *Rattus *species in South Africa and Swaziland in relation to human footprint. The different species are denoted: *R. tanezumi *(circle), *R. rattus *(square) and *R. norvegicus *(triangle) (b) Distribution of native and naturalised occurrence records of *Rattus tanezumi *in south-east Asia obtained from http://www.gbif.org. Distributional range of *Rattus tanezumi *from Global Mammal Assessment (GMA) is overlaid and indicated by grey shading.

*Rattus tanezumi *occurred in three provinces at sampling sites ranging from 446 m above sea level (a.s.l.) to 1605 m a.s.l. (Table 2; Figure 6a). *R. rattus *was recorded from all four South African Provinces sampled in this study, at altitudes ranging from 14 m to 1605 m a.s.l and from Cape Town (altitude not known) in a previous study [4]. Although *R. norvegicus *was only recorded in the Gauteng and KwaZulu-Natal Provinces, it had the broadest altitudinal range occurring at sites ranging from 6 m to 1693 m a.s.l. *Rattus tanezumi *and *R. rattus *occurred sympatrically at two Limpopo Province sites (Giyani and Tshilimbani) and at three sites in Gauteng Province (Figure 6a), *viz*. Moreleta Park (a suburb of Pretoria) and in Tembisa and Hammanskraal which are both townships incorporated within the greater metropolitan areas of Johannesburg and Pretoria, respectively. The three invasive *Rattus *species occurred sympatrically at a single site, Tembisa, which like Hammanskraal is a peri-urban township of Gauteng Province that incorporates areas of informal human settlement. Intra-specific diversity was also highest in townships within the major metropolitan areas of Durban, Pretoria and Johannesburg. Two *R. norvegicus *haplotypes (RN01 and RN02) were recovered from four Cato Crest specimens, three *R. rattus *haplotypes (RR03, RR04 and RR05) were identified from six Hammanskraal individuals and for Tembisa two haplotypes (RT01 and RT02) were identified from the four *R. tanezumi *individuals, two haplotypes (RR01 and RR03) from three *R. rattus *individuals and two haplotypes (RN01 and RN12) from four *R. norvegicus *individuals. This corresponds to a total of six *Rattus *haplotypes from just 11 specimens in Tembisa and to four *Rattus *haplotypes from the 18 individuals in Hammanskraal.

Analyses based on partial cyt *b *sequences and inclusive of *R. rattus *from diverse western Indian Ocean islands, Africa and from Guadeloupe (Lesser Antilles, Caribbean Sea) revealed that the RR01 haplotype also occurs on Grande Comore, Tanzania and Mozambique and that this haplotype is sister to a clade containing haplotypes from India, Yemen and Indonesia (see additional file 3, Figure S3). South African haplotype RR03 was unrelated to any of the presently-available *R. rattus *cyt *b *sequences, whilst the lineage containing South African RR04 and RR05 haplotypes also included two specimens from Cape Town in South Africa [4] and specimens from New Zealand, Japan, Oman, Guadeloupe, Senegal, Reunion Island and Madagascar (see additional file 3, Figure S3). The additionally-available partial *R. tanezumi *data for specimens from Laos and Indonesia revealed the existence of multiple *R. tanezumi *lineages and identified 16 haplotypes (Figure 2); the South African haplotypes RT-01 and RT-02 were, however, unrelated to any of these, being separated by at least nine mutational steps from any other haplotype.

**Figure 7:**
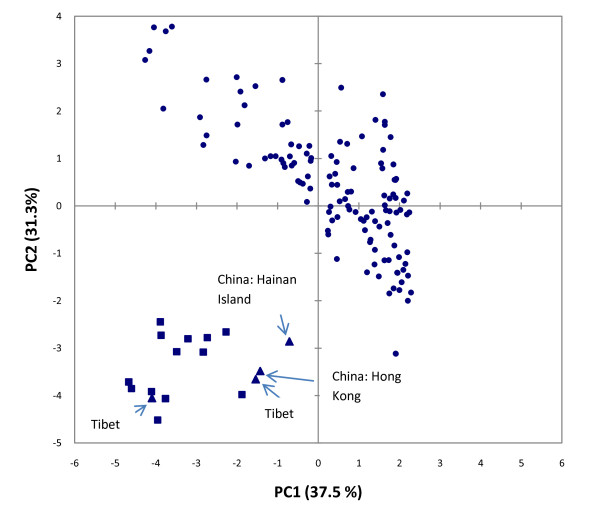
**Principal component analysis (PCA) of eight bioclimatic variables, altitude and 'human footprint' in a sample of naturalised (circles), native (triangles) and invasive South African (squares) records of *Rattus tanezumi *occurrence**.

Our sample of public records of occurrence was strongly biased towards the presumed naturalised range of the species in the Philippines and Indonesia and included just four localities in China and Tibet occurring within the probable native range of this species which is likely to be restricted to Myanmar, northern Indochina (northern Laos, central to northern Vietnam, northern Thailand) through to southern China (K. Aplin, pers. comm.). With the exception of the four northern localities in Tibet and China (including Hong Kong and Hainan Island), specimens occurring in the naturalised range of *R. tanezumi *encompass a climatic space that is somewhat distinct from that occupied by *R. tanezumi *in South Africa and Swaziland (Figure 6 & Figure 7). As very few public records were available for the assumed native distribution, we expected the model to show a measure of under-prediction of both native+naturalised and target (predicted invasive) niches. Based on examination of eigenvector loadings of variables from the PCA (not shown), the separation of the South Africa, Chinese and Tibetan records on the second principal component (PC2) is influenced strongly by a contrast between temperature variables (high positive loading for mean average temperature and minimum monthly temperature) and two variables relating to seasonality of temperature and precipitation (which showed high negative loadings on PC2; Figure 7). Thus, the above-mentioned localities (corresponding to the invasive range of South Africa and the presumed original native range in Asia) are distinguished by low mean and minimum temperatures and pronounced seasonality compared to records from the naturalised range of the species in the Philippines and Indonesia.

**Figure 8:**
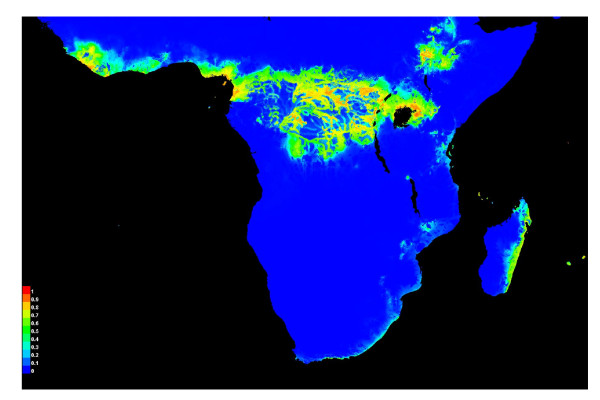
**MaxEnt model showing predicted geographical distribution of the invasive range of *Rattus tanezumi *in sub-Saharan Africa, based on the naturalised range**.

MaxEnt analyses using combined naturalised and native distribution points and five environmental variables (four bioclimatic variables and human footprint) predicted the potential invasive occurrence of *R. tanezumi *in Africa to be mostly limited to the equatorial belt and the Ethiopian Rift (Figure 8). As indicated above, this model most likely reflected a degree of under-prediction of the potential invasive distribution in Africa. The MaxEnt algorithm converged after 840 iterations with a regularized training gain of 2.03. Model performance as assessed by AUC was 0.969 for the training AUC and 0.927 for the test AUC, indicating efficient classification of suitable *versus *unsuitable habitats. Bio4 (temperature seasonality) explained most of the variation (52.2%), and it was also the variable with the highest individual gain (having the most useful information in itself). However, human footprint was the environmental variable that decreased the overall gain of the model most when omitted indicating that it contains information that is not present in the other variables. High habitat suitability of *R. tanezumi *was associated with low values of temperature seasonality and high values of annual precipitation and human influence.

## Discussion and Conclusions

The presence of *R. tanezumi *in South Africa was confirmed using a molecular and cytogenetic characterisation approach and increases the number of invasive, commensal *Rattus *species, from two to three. Haplotype diversity was low for all three invasive *Rattus *species in South Africa with just two haplotypes identified for *R. tanezumi *(RT01 and RT02), seven for *R. rattus *(RR01, RR03, RR04, RR05, RR06, RR10 and RR11) and five for *R. norvegicus *(RN01, RN02, RN03, RN04 and RN12). The cyt *b *gene phylogeny recovered three discrete monophyletic lineages for *R. rattus*, two for *R. norvegicus *and one for *R. tanezumi*, with each lineage being indicative of a separate introduction. Thus, unlike invasive *R. rattus *in Madagascar which rapidly diversified and expanded its range following what appeared to be a single introduction [33,4], cyt *b *sequence analyses of *Rattus *species from South Africa indicate that, with the exception of *R. tanezumi*, at least three *R. rattus *and two *R. norvegicus *introductions have occurred (Figure 1 and additional file 3, Figure S3). A star-like radiation of haplotypes, such as that observed for Madagascar [33,4] was only noted for one *R. norvegicus *lineage and for one *R. rattus *lineage, indicating that these two lineages most likely represent the earliest introductions to South Africa.

Comparison of South African *Rattus *sequence data with available homologous data for conspecifics revealed that two of the six *Rattus *lineage introduced to South Africa are unrelated and distinct from all other available haplotypes. In particular, the origin of the cryptic *Rattus *species, *R. tanezumi *remains obscure despite the availability of data from a number of localities within its native and naturalised ranges (Figure 2) as does the origin of the *R. rattus *haplotype RR03. The remaining two *R. rattus *haplotypes recovered from South Africa were quite widespread in Africa and its Indian oceanic islands [4]. Haplotypes RR04 and RR10 from South Africa, RR05 from South Africa and Guadeloupe, H39 from Reunion Island and RR07 from Japan, linked by one mutational step each to the RR06 haplotype which also occurs in New Zealand, Senegal, Madagascar and to two specimens from Cape Town, South Africa (Figure 3). Similarly, the RR01 haplotype identified in South Africa and which also occurs in Tanzania, Mozambique and Isle Grande Comore, linked to RR02 from Indonesia by four mutational steps (see additional file 2, Figure S2) with high levels of support (Figure 1). Common ancestry between wild-caught South African *R. norvegicus *haplotype RN12 and haplotype RN13 was indicated by the single mutational step separating these haplotypes (Figure 4) and high levels of bootstrap support (Figure 1). Of interest is that both *R. norvegicus *lineages in South Africa revealed links with laboratory strains, *viz*. RN01 is represented by BN/SsNHsdMCW, whilst the T2DN/Mcwi strain which corresponds to haplotype RN13 and is sister to RN12, was also identified in a wild-caught rat from Japan.

All three *Rattus *species were sampled in the Gauteng and KwaZulu-Natal Provinces of north-central and central-eastern South Africa, respectively, but *R. norvegicus *has to date, not been found in Limpopo Province in northern South Africa (Table 2; Figure 6a). Although the distributional range of *R. norvegicus *does not appear to extend as far north as the other *Rattus *species (Figure 6a), its presence at inland sites in Gauteng Province is significant as previous records indicated that this species was restricted to coastal areas of South Africa [22]. As *R. norvegicus *is morphologically, readily distinguished from *R. rattus*, and *R. tanezumi*, on its distinctly large overall body size alone, it is likely that its presence in the interior of the country represents a recent incursion and explains why co-occurrence of all three species has only been found at one South African site thus far. As viable hybridisation has been shown to occur between *R. rattus *and *R. tanezumi *[29] it is likely that some degree of introgression is occurring at the five sites at which these species were found to co-occur in South Africa, and should be investigated further using nuclear and mitochondrial gene characterisation in combination with karyotyping.

MaxEnt ecological niche modelling based on the combined naturalised and native range of *R. tanezumi *failed to predict the known (and presumably recent) invasive range of the species in the interior of South Africa. Although this could be partly explained by under-prediction due to the incomplete sampling of the presumed original native range (only four records available) compared to the naturalised range, it is also conceivable that factors other than climate have facilitated the spread of the species in South Africa. If it could be shown that South African populations originated from the more temperate, presumed-native range of the species in Asia, niche similarity could explain its presence in South Africa (as demonstrated by the proximity of the records in the bioclimatic space in Figure 7). Nevertheless, even within south-eastern Asia the species has spread from its original native range in more temperate climates, to invade tropical and equatorial areas extending into the Philippines and Indonesia, suggesting the importance of factors other than climate. Given the highly commensal nature of *R. tanezumi*, it is noteworthy that records of this species in South Africa largely coincided with nodes of high human influence (human footprint; Figure 6a). Given the extent of historical shipping trade between South Africa and the Far East, the accidental introduction of *R. tanezumi *is easily explained, where after this species has apparently exploited the commensal niche, using major transport routes to disperse as far inland as the commercial-industrial hub of Gauteng Province. The further dispersal of this species to the northern Limpopo Province could be explained by the presence of the N1 highway and dense rural populations and markets in the Venda region, (where *R. tanezumi *was first recorded in South Africa).

The origins of the *Rattus *species introduced into South Africa could not be accurately pin-pointed and will remain obscure until a comprehensive database that includes all invasive *Rattus *species from throughout their native and introduced geographical ranges, becomes available. In this regard, this study has made a valuable contribution to documenting feral *R. norvegicus *diversity and distribution, as only a limited number of complete cyt *b *haplotype sequences were available prior to this study (Table 1). The additional data generated for the five South African *R. norvegicus *haplotypes and for three wild-caught animals from Vietnam and Indonesia, contribute to wild-caught *R. norvegicus *haplotype diversity data and would benefit from similar studies in all regions in which this invasive species occurs. The number of full-length reference cyt *b *gene sequences for *R. rattus *has also increased. Despite a larger number of reference haplotype sequences being available from the naturalised and native ranges of *R. tanezumi *[8] and *R. rattus *[4], these were insufficient to provide insight into the likely origin of the *R. tanezumi *lineage introduced into South Africa, which differed by a minimum of nine mutational steps from all available sequences, or for the *R. rattus *RR03 lineage. Sampling and sequencing efforts need to be intensified globally for these three invasive *Rattus *species in order to map migration pathways more accurately and to assess factors influencing and limiting their co-occurrence. Apart from contributing to general small mammal studies in Africa, the present study may have implications in epidemiological, agricultural, biological conservation, and invasion biology research associated with cryptic invasive rodents. It is unlikely that it is only in South Africa that the presence of *R. tanezumi *has been obscured because of its morphological similarity to *R. rattus*. Genetic monitoring was crucial for detecting this cryptic invasive species that silently became established throughout much of the established range of competing congeners, and is an approach that should be considered in all areas to which members of the *R. rattus *species complex have been introduced.

## Competing interests

The authors declare that they have no competing interests.

## Authors' contributions

Sample and field data collection: FK, EvM, PJT. Nucleotide sequence data generation: ADSB, HB, DN, MEM, JML, SD. Molecular analyses: ADSB, PvH. Karyotyping: DN, GC. GIS analyses: PJT. Drafted the manuscript: ADSB. All authors read, approved and contributed to the manuscript.

## Supplementary Material

Additional file 1 Table S1: Eigenvectors from first three principal components (PC) analysis of eight bioclimatic variables in 134 native and 13 invasive records of occurrence. Click here for file

Additional file 2 **Figure S2: Median-joining networks of cytochrome *b *(cyt *b*) haplotypes for each of the three *Rattus *species in South Africa (a) *Rattus tanezumi *(full-length), (b) *Rattus rattus *(full-length).** The circle size of South African *Rattus *haplotypes is proportional to the frequency of the haplotype. Haplotype colour coding is consistent with that used in Figure 1, *viz*. Yellow = south Africa (This study), Green = Africa (Genbank), Grey = Outside Africa (Genbank). Each of the mutational steps separating haplotypes is indicated in blue and corresponds to the relevant position in the cyt *b *gene, whilst black nodes correspond to median vectors. Haplotype numbers are the same as those provided in Table 1. Click here for file

Additional file 3 **Figure S3: Neighbor-joining tree depicting *R. rattus *haplotype relationships inferred from a homologous 1043 bp region of the mitochondrial cytochrome *b *gene.** Nodal support values, expressed as a percentage and ≥ 55 are indicated next to the relevant nodes and are based on 1,000,000 bootstrap replications. The tree was rooted with *R. tanezumi*, haplotype RT01. Square brackets are used to indicate haplotypes defined in the Tollenaere *et al*. [4] study. The haplotypes identified on the basis of complete cyt *b *sequence data, and assigned in this study are indicated in bold and denoted RR01-RR10. Click here for file
