# Supplementary material for: Genetic monitoring detects an overlooked cryptic species and reveals the diversity and distribution of three invasive Rattus congeners in south Africa
Source: BMC Genet. 2011 Feb 16;12:26. doi: 10.1186/1471-2156-12-26 (PMC3055845; doi:10.1186/1471-2156-12-26)
Supplement: Additional file 1 — Table S1: Eigenvectors from first three principal components (PC) analysis of eight bioclimatic variables in 134 native and 13 invasive records of occurrence. [file 1471-2156-12-26-S1.DOC]

**Additional file 1**

**TABLE S1**

| **Variable** | **PC1** | **PC2** | **PC3** |
| --- | --- | --- | --- |
| Annual precipitation (Bio12) | 0.280 | -0.319 | 0.509 |
| Precipitation of wettest month (Bio13) | 0.169 | -0.046 | 0.702 |
| Precipitation of driest month (Bio14) | 0.300 | -0.457 | -0.148 |
| Precipitation seasonality (Bio15) | -0.278 | 0.369 | 0.461 |
| Mean annual temperature (Bio1) | 0.426 | 0.413 | -0.051 |
| Temperature seasonality (Bio4) | -0.441 | 0.223 | 0.076 |
| Maximum temperature of warmest month (Bio5) | 0.275 | 0.534 | -0.060 |
| Minimum temperature of coldest month (Bio6) | 0.523 | 0.214 | -0.034 |
