# Supplementary material for: Genetic monitoring detects an overlooked cryptic species and reveals the diversity and distribution of three invasive Rattus congeners in south Africa
Source: BMC Genet. 2011 Feb 16;12:26. doi: 10.1186/1471-2156-12-26 (PMC3055845; doi:10.1186/1471-2156-12-26)
Supplement: Additional file 2 — Figure S2: Median-joining networks of cytochrome b (cyt b) haplotypes for each of the three Rattus species in South Africa (a) Rattus tanezumi (full-length), (b) Rattus rattus (full-length). The circle size of South African Rattus haplotypes is proportional to the frequency of the haplotype. Haplotype colour coding is consistent with that used in Figure 1, viz. Yellow = south Africa (This study), Green = Africa (Genbank), Grey = Outside Africa (Genbank). Each of the mutational steps separating haplotypes is indicated in blue and corresponds to the relevant position in the cyt b gene, whilst black nodes correspond to median vectors. Haplotype numbers are the same as those provided in Table 1. [file 1471-2156-12-26-S2.DOC]

**Additional file 2 (Fig. S2)**

1. *Rattus tanezumi* (full-length dataset; 1140 bp)

1. *Rattus rattus* (full-length dataset; 1140 bp)
