# Supplementary material for: Genetic monitoring detects an overlooked cryptic species and reveals the diversity and distribution of three invasive Rattus congeners in south Africa
Source: BMC Genet. 2011 Feb 16;12:26. doi: 10.1186/1471-2156-12-26 (PMC3055845; doi:10.1186/1471-2156-12-26)
Supplement: Additional file 3 — Figure S3: Neighbor-joining tree depicting R. rattus haplotype relationships inferred from a homologous 1043 bp region of the mitochondrial cytochrome b gene. Nodal support values, expressed as a percentage and ≥ 55 are indicated next to the relevant nodes and are based on 1,000,000 bootstrap replications. The tree was rooted with R. tanezumi, haplotype RT01. Square brackets are used to indicate haplotypes defined in the Tollenaere et al. [4] study. The haplotypes identified on the basis of complete cyt b sequence data, and assigned in this study are indicated in bold and denoted RR01-RR10. [file 1471-2156-12-26-S3.DOC]

**Additional file 3 (Fig. S3)**

**RR01** South Africa (N=16); Tanzania, Mozambique, Comores

Comores [H24, H28]

Comores [H26]

India [H4]; Comores [H27

**RR02** Indonesia

Yemen [H14]

Yemen [H13]

Oman [H6]

India [H5]

**RR04** South Africa (N=8)

**RR06** Madagascar (N=1), South Africa (N=2), Senegal (N=2); New Zealand (N=1)

Reunion [H39]

**RR10** South Africa (N=1)

**RR05** South Africa (N=1); Guadeloupe (N=2)

**RR11** South Africa (N=1)

**RR07** Japan

Oman [H12]

**RR03** South Africa (N=9)

**RR08** India

Tanzania [H17]

Oman [H10, H11]

Oman [H9]

Ethiopia [H16]

Mayotte [H29]

Madagascar, Mozambique [H20, 21, 30, 32, 36]

Madagascar [H33]

Madagascar [H37]

Madagascar [H34]

**RR09** Oman

Madagascar [H35]

Ethiopia [H15]

India [H1]

Oman [H8]

India [H2]

**70**

**63**

**54**

**63**

**83**

**62**

**71**

**75**

**77**

**66**

0.001
